# Diffuse reflectance spectroscopy reveals heat stress-induced changes in hemoglobin concentration in chicken breast

**DOI:** 10.1038/s41598-021-83293-y

**Published:** 2021-02-11

**Authors:** Sina Dadgar, Elizabeth Greene, Ahmed Dhamad, Barbara Mallmann, Sami Dridi, Narasimhan Rajaram

**Affiliations:** 1 Department of Biomedical Engineering, University of Arkansas, Fayetteville, AR 72701 USA; 2 Department of Poultry Science, University of Arkansas, Fayetteville, AR 72701 USA

**Keywords:** Optical spectroscopy, Animal physiology

## Abstract

Heat stress (HS) is devastating to the poultry industry due to its adverse effects on animal well-being and performance. The effects of heat stress are typically measured using a portable i-STAT blood analyzer that quantifies circulatory hemoglobin concentration and other blood chemistry parameters. Here, we used diffuse reflectance spectroscopy (DRS) as a novel non-invasive method to directly determine changes in hematological parameters in the breast tissues of live heat-stressed broilers. Three-week-old male broilers were randomly subjected to two environmental conditions (thermoneutral, TN, 24 °C vs. cyclic heat stress, HS, 35 °C, 12 h/day). Optical spectra were acquired using DRS to monitor breast hemoglobin (Hb) concentration and vascular oxygen saturation (sO_2_) at three time points: at baseline prior to heat stress, 2 days, and 21 days after initiation of HS. While i-STAT did not demonstrate a discernible change due to HS in circulatory hemoglobin, DRS found a significant decrease in breast Hb and sO_2_ after exposure to chronic HS. The decrease in sO_2_ was found to be due to a decrease in oxygenated hemoglobin concentration, indicating a large increase in oxygen consumption in heat-stressed broilers. Our results demonstrate that DRS could potentially be used to study the effects of HS directly in specific organs of interest, such as the breast and thigh, to improve meat quality.

## Introduction

Poultry meat is a mass consumer product and one of the main efficient food sources worldwide for billions of people. By 2024, it is expected that the per capita consumption of poultry meat in the US will reach 49.3 kg/capita/year^1^. However, the future of the poultry industry is in jeopardy with increases in heat waves and global temperature warming^2,3^. Heat stress (HS) has devastating consequences to poultry industry and negatively impacts animal welfare, growth performance, and egg quality^4–6^. Heat stress occurs when heat production by animal body exceeds its heat dissipation capability, which leads to depression in feed intake, reduction in body weight, and hence, increased mortality rate^7^. To increase heat loss, birds divert blood to the periphery (skin) which results in a hypoxia-like state in the internal organs such as the breast muscle^8^. Several studies have utilized the i-STAT portable blood analyzer to investigate the effects of heat stress on blood parameters. Wang and colleagues studied the effects of acute and chronic heat stress on two genetically distinct inbred chicken lines and found that a number of blood parameters, namely PO_2_, TCO_2_, and HCO_3_ could serve as biomarkers to improve heat tolerance in poultry^9^. A recent study by Rowland et al. used i-STAT measurements to demonstrate that several blood gas parameters are heritable in response to acute or chronic heat exposure^5^. Exposure to acute heat stress was also found to result in decreased eggshell weight, likely due to a reduction in blood iCa levels^6^. By virtue of sampling whole blood parameters, the i-STAT provides quantitative metrics that can be used to determine overall animal wellbeing. Here, we utilize a noninvasive optical technology for real time, in vivo investigation of hemoglobin-based parameters in the chicken breast, an important part of the animal that contributes to the poultry industry.

Diffuse reflectance spectroscopy (DRS) is an optical fiber-based technique that uses ‘source’ optical fibers to deliver low-power non-ionizing broadband white light to the tissue surface and ‘detector’ optical fibers to collect the diffusely reflected light from tissue^10^. The source and detector fibers are typically separated from each other, with the source-detector separation distance (SDSD) determining the sampling depth within tissue^11^. This diffusely reflected light carries the spectral signatures of tissue components that interacted with the light. These ‘interactions’ consist of (1) elastic scattering (no loss of energy but a change in direction) caused by refractive index mismatch as light travels within different cellular and tissue constituents, such as cell nuclei, mitochondria and collagen, and (2) absorption, primarily by oxygenated and deoxygenated hemoglobin in blood vessels. Depending on tissue type, other major absorbers in this wavelength range used in this study (480–600 nm) include melanin (skin) and beta-carotene (breast). Inelastic Raman scattering and fluorescence emission from tissue are other forms of light-tissue interaction; however, the observation of these interactions in tissue requires the use of monochromatic light sources for excitation and highly sensitive detectors. A detailed description of these light tissue interactions can be found in the literature^12^. By analyzing the diffusely reflected light from tissue using analytical or quantitative models of light-tissue interaction, it is possible to determine the scattering and absorption properties of interrogated tissue, and extract meaningful information regarding tissue scattering, hemoglobin concentration, and vascular oxygen saturation. Specifically, the distinct absorption spectra of oxygenated (HbO_2_) and deoxygenated hemoglobin (dHb) allow the quantification of total hemoglobin concentration (THb = HbO_2_ + dHb) and vascular oxygen saturation (sO_2_ = HbO_2_/THb) in the tissue sampling volume. Previous work by us and others have demonstrated that vascular oxygenation measured using DRS is correlated with immunohistochemical measures of hypoxic fraction^13^ and microelectrode based measures of tissue oxygenation (pO_2_)^14^. One of the foremost applications of DRS has been in the field of cancer as a potential complementary tool to histopathology. These applications have spanned early cancer detection in several organs including skin^15^ and breast^16^, identification of surgical margins^17^, and monitoring response to therapy^18,19^. The underlying premise of these applications is that the major sources of tissue scattering and absorption are also molecules that undergo significant changes during disease progression or in response to external stimuli; therefore DRS can provide a noninvasive and nonionizing ‘optical biopsy’ of tissue. The simplicity of the tool also lends itself to potentially repeated measurements on the same tissue, thus providing a continuous time-lapse of changes in tissue rather than a single snapshot.

Using DRS, we have previously shown that circulatory and breast muscle oxygen homeostasis is deregulated in chickens with woody breast (WB) myopathy compared to healthy counterparts^20^. Given the sensitivity of DRS to changes in vascular oxygenation and the relevance of tissue hypoxia in chicken breast pathology, we sought to use DRS to investigate the effects of HS on chicken breast hemoglobin concentration and vascular oxygen saturation. In the following sections, we describe the diffuse reflectance method used to measure optical spectra, the heat stress protocol, and the analysis of the optical spectra. Optical spectra from the breast of heat-stressed and control chicken were recorded at three time points—prior to heat stress, 2 days after heat stress, which we defined as acute HS and 21 days after heat stress, which we defined as chronic HS. Vascular oxygen saturation and hemoglobin concentration were quantified from these spectra. Results from this study and a discussion of the data as well as possible implications are presented in subsequent sections.

## Materials and methods

### Animal storage and handling

**Figure 1:**
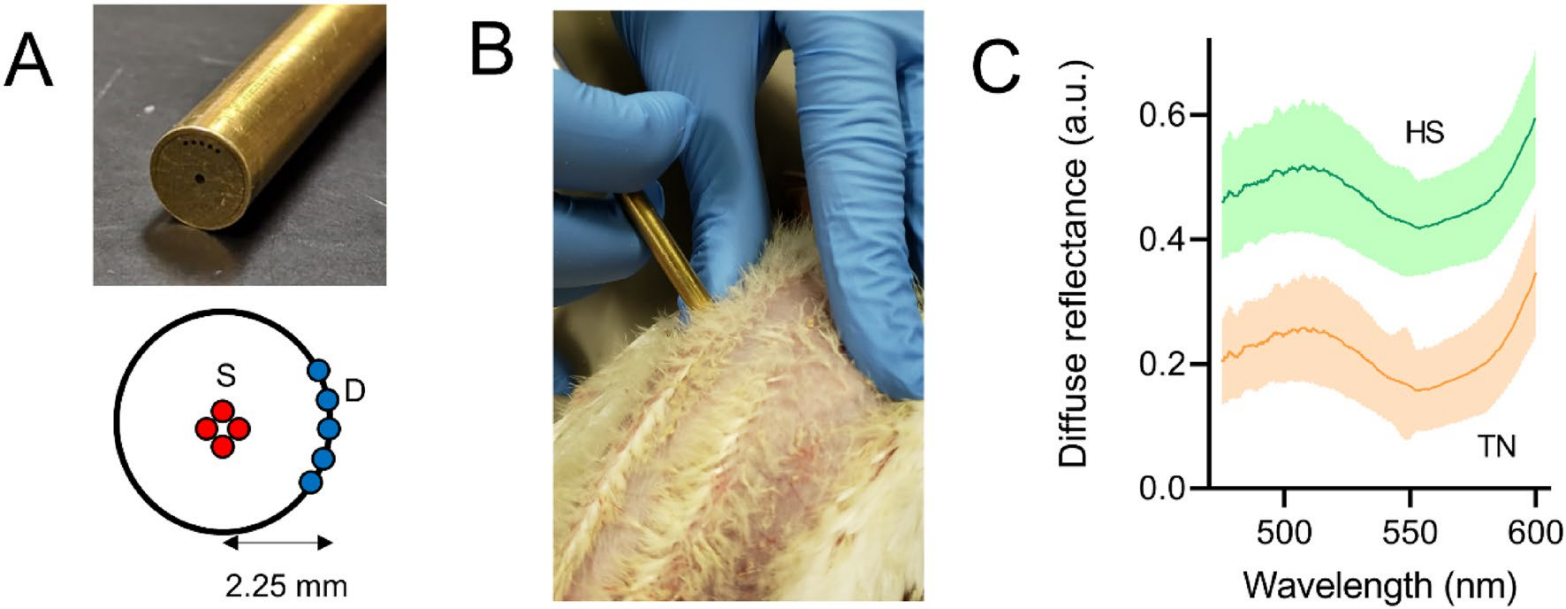
(**A**) Optical probe design with a source detector separation distance of 2.25 mm. S and D respectively illustrate the source and detector fibers. (**B**) Performing optical spectroscopic measurements from the breast of a chicken. Animals were held upside down during the process of optical data acquisition. (**C**). Mean diffuse reflectance spectra with semitransparent shadow representing standard deviation. Data acquired from TN (orange) and HS (green) animals. Spectra are offset for visualization purposes only.

The studies with chickens were approved by the University of Arkansas Institutional Animal Care and Use Committee (IACUC Protocol #16084). Three-week-old broiler chickens (Cobb 500, n = 16) were randomly divided into cyclic heat stress (HS, 35 °C for 12 h/day) and thermoneutral conditions (TN, 24 °C) and were given ad libitum access to food and clean water. We collected optical data and blood samples at three time points: before exposure to heat-stress (pre-HS), 2 days after initiation of HS (Acute-HS), and 21 days after HS (Chronic-HS). First, 10 mL of blood sample were collected from femoral artery from each animal. This was followed by optical data acquisition from chicken breast while animals were held upside down (Fig. 1B). All experiments were performed in accordance with relevant guidelines and regulations.

### Diffuse reflectance spectroscopy

Our spectroscopic system consists of a halogen lamp (HL-2000, Ocean Optics, Dunedin, Florida) as light source, a USB spectrometer (spectral resolution: 0.22 nm; Flame, Ocean Optics) for spectral acquisition, and a hand-held bifurcated probe for light delivery and collection. Figure 1A illustrates the tip of our probe. Covered with surgical stainless-steel jacketing, the common end of the probe houses illumination and collection fibers. The probe head is 6.4 mm in diameter and consists of 4 illumination fibers (diameter = 200 μm; NA = 0.22), located at the center of the metal ferrule, and 5 detection fibers, located at 2.25 mm away from the center (FiberTech Optica, Ontario, Canada). We have determined this probe samples light from a depth of approximately 1.25 mm in the spectral range of 450–600 nm^11^. The proximal ends of the probe are terminated with subminiature version A (SMA) connectors which allowed their secure attachment to the lamp and the spectrometer. The optical fibers are arranged in a linear manner at the spectrometer end to ensure that the fibers are aligned with the linear slit. The entrance aperture of the spectrometer contains a 200 µm slit. This slit width was selected to match the diameter of our optical fibers to ensure all the light from the fibers is collected at the CCD. Perfect alignment of the detector end with the linear slit is confirmed by adjusting the fiber until maximum intensity is detected at the CCD. At the light source, the fibers are arranged in the same circular manner as the tissue end of the probe. Data acquisition was simplified by using a foot pedal controlled by a custom LabVIEW (National Instruments, Austin, TX) program. The integration time of the spectrometer was set to 100 ms for all spectroscopic measurements to ensure that diffusely reflected light from the tissue (chicken breast) did not saturate the 16-bit CCD of the spectrometer (max pixel intensity of 2^16^). This integration time was also short enough to enable spectral acquisition from multiple locations on the chicken breast and minimize possible motion artifacts. Spectral data acquisition from each chicken was completed in about 30 s. We collected spectra from a minimum of 15 different locations on the chicken breast in the wavelength range of 475 to 600 nm. The spectral range was narrowed to this window because eliminating wavelengths below 450 nm limits the effect of pigment packaging, (i.e. underestimation of hemoglobin absorption)^21^. In addition, a short wavelength range reduces partial volume effects and ensures interrogation of a narrow range of depth in tissue. Finally, this wavelength range has been shown to be sufficient for quantifying vascular oxygenation that is inversely correlated with tissue hypoxic fraction^13^. Spectra acquired from chicken breast were background subtracted to eliminate ambient light. This was performed by blocking light entry from light source into the tissue using an unmounted 2″ × 2″ absorptive filter with optical density of 6.0 (NE260B, Thorlabs, Newton, NJ). On each day prior to any optical measurement from animals, reflected light intensity of an 80% reflectance standard (SRS-80-010; Labsphere, North Sutton, New Hampshire) was acquired to calibrate for daily variations in light throughput. This was performed by securing the optical probe using an optical post clamp (RPS—Thorlabs, Newton, NJ) and positioning it at the same pre-determined height from the surface of reflectance standard. Diffusely reflected light from each chicken breast was calculated according to equation below:

1 $${\text{R}} = \frac{{{{\text{I}}_{\text{t}}} - {{\text{I}}_{{\text{tbgd}}}}}}{{{{\left( {{{\text{I}}_{\text{s}}} - {{\text{I}}_{{\text{sbgd}}}}} \right)} / {0.8}}}}$$

where R, ${\mathrm{I}}_{\mathrm{t}}$, ${\mathrm{I}}_{\mathrm{tbgd}}$, ${\mathrm{I}}_{\mathrm{s}}$, and ${\mathrm{I}}_{\mathrm{sbgd}}$ respectively are reflectance, tissue light intensity, tissue dark noise, standard light intensity, and standard dark noise. The denominator is divided by 0.8 because we use an 80% reflectance standard. Average diffuse reflectance spectra from the breast of the HS and TN chickens are presented in Fig. 1C. The spectra shown here are offset for visualization purposes only and indicate that there are no visible group dispersions within DRS spectra of two groups. However, data analysis was performed on original raw data.

### Quantification of tissue optical properties

We have previously developed a look table (LUT)-based inverse model to fit the measured spectra and extract wavelength-dependent absorption and scattering properties of tissue. This empirical model has been described previously^22^ and validated for a range of different source detector separations (SDSDs), multiple absorbers, and different scatter sizes^11^. Briefly, diffuse reflectance spectra are acquired over a wide range of wavelengths from a matrix of tissue-simulating phantoms of known scattering and absorption properties to enable the construction of a lookup table of reflectance values as a function of scattering and absorption (corresponding to each wavelength). We used a single-layer model and thickness was not considered while constructing the LUT model. Vishwanath et al*.* have shown that for wavelengths greater than 450 nm, the optical properties determined using a single layer model do not differ from a multi-layer assumption^23^. The two primary variables in constructing the LUT were the absorption coefficient (μ_a_) and reduced scattering coefficient (μ_s_′). For the inverse model, we constrained scattering to follow a negative power-law dependence on wavelength^24^:

2 $$\upmu^{ \prime}\left( \uplambda \right) = \upmu _{\text{s}}^{\prime}\left( {{\uplambda _0}} \right) \cdot {\left( {{\raise0.7ex\hbox{$\uplambda $} \!\mathord{\left/ {\vphantom {\uplambda {{\uplambda _0}}}}\right.\kern-\nulldelimiterspace} \!\lower0.7ex\hbox{${{\uplambda _0}}$}}} \right)^{ - {\text{B}}}}$$

with ${\uplambda }_{0}=600\mathrm{ nm}$ as a reference point where light absorption is minimum. We assumed light absorption to be a linear sum of light absorbing chromophores, namely oxygenated and deoxygenated hemoglobin, as well as absorption from skin. In the spectral range of 475–600 nm, we calculated ${\upmu }_{\mathrm{a}}$ as:

3 $${\upmu }_{\mathrm{a}}\left(\uplambda \right)=\left[\mathrm{Hb}\right][\mathrm{\alpha }{\upsigma }_{{\mathrm{HbO}}_{2}}\left(\uplambda \right)+\left(1-\mathrm{ \alpha }{)\upsigma }_{\mathrm{dHb}}\left(\uplambda \right)\right]+\left[\mathrm{skin}\right]\mathrm{skin}(\uplambda )$$

where [Hb] and [skin] respectively are concentrations of total hemoglobin and skin absorbing chromophores (mg/mL). α is vascular oxygen saturation which represents the ratio of oxygenated (HbO_2_) to total hemoglobin concentration [Hb]. The fixed absorption parameters, extinction coefficients of oxygenated hemoglobin (${\upsigma }_{{\mathrm{HbO}}_{2}}$), deoxygenated hemoglobin (${\upsigma }_{\mathrm{dHb}}$), and skin were obtained from an online database^25^. The inclusion of skin absorption is done only to account for the additional absorption through the skin layer and its effect on the diffuse reflectance spectrum. Because the shape of skin absorption is similar to the power law nature of Mie scattering in tissue, we constrain the bounds of skin absorption over a narrow range. The range of μ_s_′ and μ_a_ in the LUT were 1.93–10.92 cm^−1^ and 0–23.5 cm^−1^, which represents the range of values over which the LUT can accurately determine optical properties of tissue. To fit the model to the acquired spectra, we implemented a non-linear optimization fitting routine for extracting optical properties. Five ‘free parameters’ (${\upmu }_{\mathrm{s}}{^{\prime}}({\uplambda }_{0})$, B, [Hb], α, and skin) from Eqs. (2) and (3) were assigned with certain initial values. Since the other parameters were known a priori (e.g. ${\upsigma }_{\mathrm{dHb}}$), wavelength-dependent ${\upmu }_{\mathrm{a}}$ and ${\upmu }_{\mathrm{s}}{^{\prime}}$ were calculated and fed into our empirically built LUT to extract their unique corresponding reflectance value. Next, the extracted reflectance spectrum was compared to the actual reflectance spectrum and the initial error (chi-square error) was calculated. Finally, this error was minimized by efficiently manipulating our free parameters (within certain upper and lower bands). The final free parameters after optimization were reported as the optical properties of a specific location on the chicken breast. The optical properties of each chicken breast were calculated as the average over multiple locations. Data analysis was performed in MATLAB (MathWorks, Natick, MA).

### i-STAT system

Circulatory hemoglobin concentration (Hb) was determined using i-STAT Alinity system (SN: 801128; software version JAMS 80.A.1/CLEW D36; Abaxis, Union City, CA) with the i-STAT CG8 + cartridge test (ABBT-03P77-25) according to manufacturer’s recommendation. i-STAT derived hemoglobin values have previously been validated in laying hens^26^.

### Statistical analysis

Repeated measures analysis of two-factor ANOVA was employed to determine statistically significant differences in Hb levels from both DRS and i-STAT readings in different treatments (NT vs. HS), time points, (pre-HS vs. Acute-HS vs. Chronic-HS) and their interactions. Time and treatment were respectively considered as within and between effects. Additionally, interactions between all effects were included in the analysis. Post-hoc Tukey HSD test were used to differentiate specific groups. All statistical analyses were performed using JMP.

## Results

**Figure 2:**
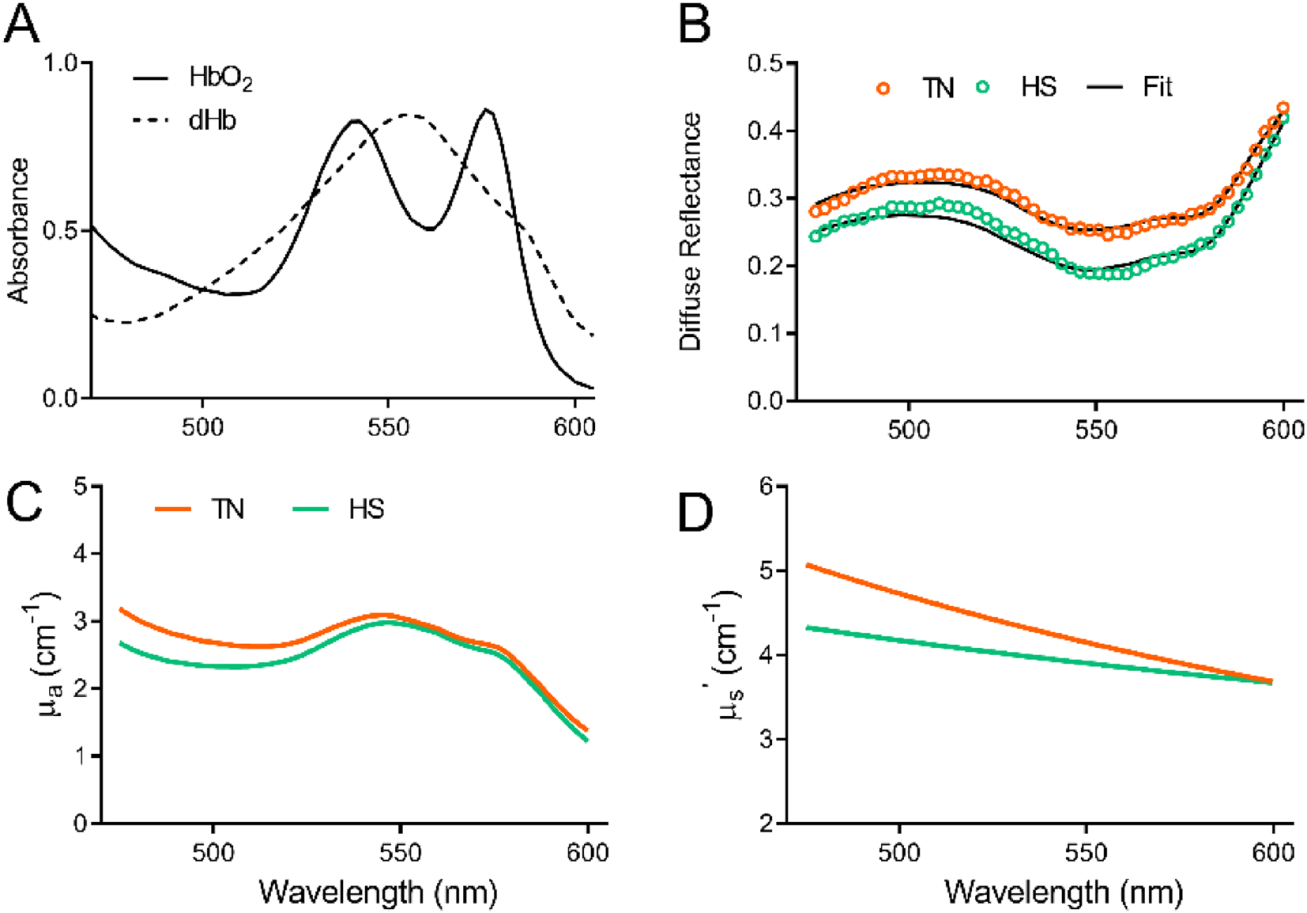
(**A**) Absorbance spectra of oxygenated and deoxygenated Hb. (**B**) Measured diffuse reflectance spectra of representative animals in TN (orange circles) and HS (red circles) and their corresponding LUT fit (black line) collected at pre-HS time point. LUT extracted absorption (**C**) and scattering (**D**) coefficients.

Figure 2A illustrates the distinct absorption spectra of oxygenated (HbO_2_—solid line) and deoxygenated hemoglobin (dHb—dashed line). While HbO_2_ has dual peaks at 542 and 576 nm, dHb has a single absorption peak at 560 nm. Figure 2B presents diffuse reflectance spectra measured from two representative animals in TN (orange circles) and HS (green circles) groups and their corresponding LUT fits (black line) at pre-HS time points. The double bands of the spectrum acquired from the animal from HS group (green line) is slightly more pronounced than the TN (orange line) group. In addition, the DRS spectrum acquired from the chicken from HS group has lower magnitude of diffuse reflectance compared with the spectrum collected from TN animal. While both spectra appear to show similar absorption profiles, with slightly more pronounced hemoglobin absorption bands in the HS spectrum (Fig. 2C), the differences in the overall magnitude of the DRS spectra are mostly driven by differences in scattering at shorter wavelengths (Fig. 2D).

**Figure 3:**
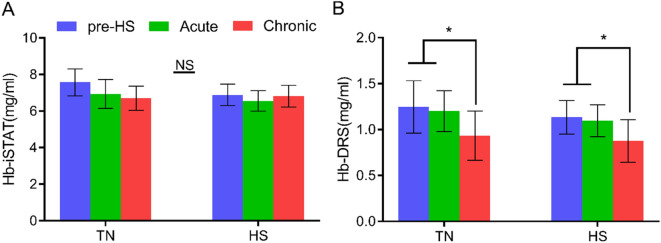
Comparison of i-STAT and DRS-based measurements. (**A**) Circulatory hemoglobin concentration detected by i-STAT. (**B**) DRS-based total hemoglobin concentration. Data are presented as mean ± SEM. Pre-HS (blue bars) indicates baseline measurements prior to heat stress, Acute-HS (green bars) indicates measurements on Day 2, and Chronic-HS (red bars) measurements are from Day 21. Two factor ANOVA analysis was performed on both datasets assessing significant differences in treatment (TN vs. HS), time (pre vs. acute vs. chronic) and their interactions. No significance (NS) was observed in i-STAT related Hb values. *Indicated p < 0.05.

To enable quantitative comparisons between the different groups, we determined the fit parameters derived from the absorption and scattering coefficients. Figure 3 shows the temporal kinetics of the concentration of i-STAT-measured Hb (Fig. 3A) and the concentration of DRS-estimated Hb (Fig. 3B). For each treatment, the data are presented as pre-HS (blue bars), Acute-HS (green bars), and Chronic-HS (red bars). Both i-STAT and optical measurements demonstrate a trend towards lower [Hb] in the control and heat stress groups. While no significant differences between groups were found in i-STAT measurements of circulatory [Hb], optically measured [Hb] in the chicken breast was significantly lower at the chronic HS time point compared with pre-HS and acute-HS in both the control and heat stressed-animals.

**Figure 4:**
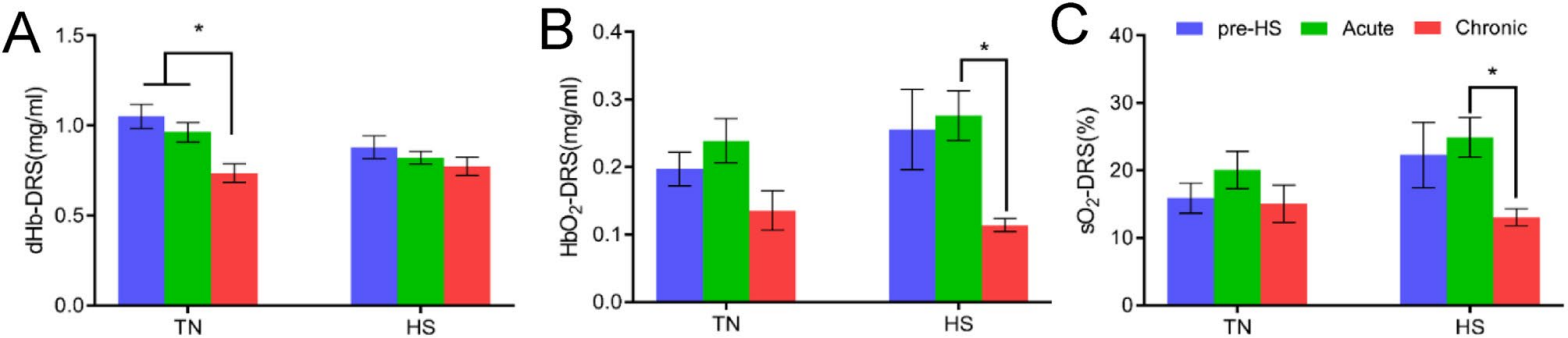
Extraction of DRS-based optical properties: (**A**) Deoxygenated hemoglobin (dHb) (**B**) Oxygenated hemoglobin (HbO_2_) and (**C**) hemoglobin oxygen saturation—sO_2_. Error bars indicate standard error of the mean.

To understand the source of observed changes in optically derived [Hb] among the treatment groups, we determined the concentrations of deoxygenated hemoglobin (dHb—Fig. 4A), oxygenated hemoglobin (HbO_2_—Fig. 4B) and vascular oxygen saturation (sO_2_—Fig. 4C) as the ratio of HbO_2_/(dhb + HbO_2_). We observed a significant reduction in [dHb] in the TN group at Day 21 (chronic HS time point) and a significant decrease in [HbO_2_] in the chronic HS group. No significant differences were found in the TN group over time. While sO_2_ in the TN group remained relatively unchanged over the 21-day period, sO_2_ in the chickens exposed to HS decreased over time. Chickens at the chronic HS time point exhibited a significantly lower sO_2_ compared with their acute HS measurements. This significant decrease in sO_2_ observed at the chronic HS time point (Day 21) is due to the significant decrease in [HbO_2_].

**Figure 5:**
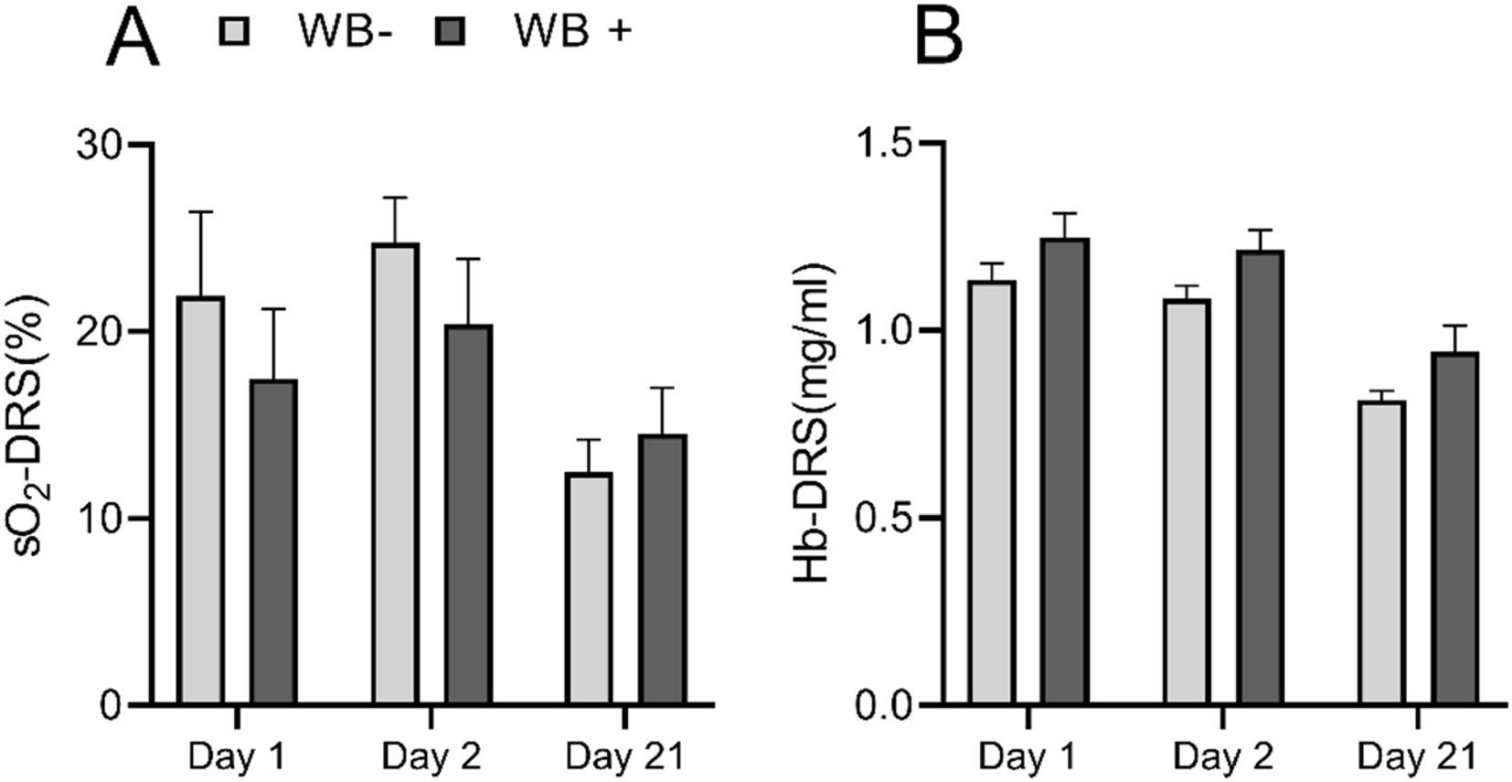
Comparison of vascular oxygen saturation and hemoglobin concentration in Woody breast (WB)-positive and negative chickens at different time points during heat stress (HS). Here, Day 2 represents the acute HS time point and Day 21 represents the chronic HS time point. Error bars represent standard error of the mean (SEM).

Select animals from both the TN and HS groups were euthanized and, after slaughter process and macroscopic processing, breast fillets were classified as woody breast positive and woody breast negative. Figure 5 illustrates temporal changes in optically determined sO_2_ (Fig. 5A) and [Hb] (Fig. 5B) in woody breast positive and negative animals. Although there were no significant differences between the two groups of chickens, we noted that WB-positive chickens presented lower oxygen saturation in comparison to WB-negative animals at the pre-HS and acute HS time points, and a higher total hemoglobin concentration at all time points.

## Discussion

Due to its non-ionizing and noninvasive nature, fiber-based diffuse optical spectroscopy has been used in a plethora of pre-clinical and clinical studies in biomedical research with the goal of earlier detection of pathological changes in tissue and hence improved human health. Over the years, such technologies have shown and continue to demonstrate great promise in identifying functional and molecular changes within tissue that precede anatomical changes. Our recent work found that measurements of vascular oxygen saturation using DRS could identify chickens with woody breast very early in their development^20^. In this study, we used DRS to investigate the effects of heat stress on the chicken breast in live chickens and compared the data with i-STAT measurements on the same chicken. DRS provides a method to measure vascular changes directly from organs of interest, such as the chicken breast or thigh. By extending spectral range of optical measurements to near infrared (NIR), optical measurements can be sensitive to measurements of lipid and water content in tissue^27^, both of which can be useful in HS studies^28,29^. On the other hand, i-STAT measurements can provide quantitative measurements of electrolytes in the sampled blood in addition to hemoglobin concentration^30^, information that DRS is incapable of providing. A combination of these two techniques can likely yield complementary information to evaluate the health status of a chicken and hence meat quality.

Our results indicate a trend towards a decrease in [Hb] in chickens that were exposed to heat stress as well as thermoneutral (control conditions). While DRS-based [Hb] was significantly lower after 21 days of exposure to heat stress, the i-STAT measurements did not show any significant changes over the 21-day period in the two groups of chickens. This could be explained by the fact that the DRS-based measurements are providing a measure of hemoglobin concentration changes at the tissue level whereas the i-STAT analyses circulatory hemoglobin concentration and provides a measure of health at a whole-body level. Previous studies have found an increase in blood [Hb], as measured with i-STAT, as chickens grew older^31,32^. To the best of our knowledge, we are not aware of other studies that have measured tissue-level [Hb], and therefore these results deserve further investigation. While the decrease in [Hb] was observed in both TN and HS groups over time, the source of this reduction appears to be different in both groups. Specifically, there was a significant decrease in [dHb] concentration in the chronic-HS time point of TN group which could possibly be due to a reduction in oxygen consumption rate (OCR). However, we did not observe such changes at the acute time point. Due to logistical reasons, we were unable to acquire optical spectra and i-STAT measurements immediately following heat stress and the earliest time point at which we acquired data was at 48 h. It is possible that any short-term changes due to heat stress manifest over the first 12 h, as determined previously by others^33,34^, and are no longer discernible by 48 h. The decrease in hemoglobin concentration in animals from HS group appears to be driven primarily by a large, significant decrease in [HbO_2_], which could indicate a large increase in oxygen consumption after exposure to HS to meet the energy needs of the muscle. Previous work by Persia and colleagues has shown that animals exposed to 4 weeks of HS had significant reduction in blood parameters of sO_2_ and pO_2_^5,6^ In addition, chronic heat stress has been shown to lead to increased generation of lactic acid and decreased pH in chicken breast and thigh muscle^35^. We believe that acquiring optical spectra at more frequent intervals would likely identify earlier time points than Day 2 and Day 21 at which heat stress-induced short-term and long-term changes are apparent.

In addition to HS and its consequences on breast physiology, our results illustrate that DRS can be used in dynamic monitoring of tissue hemoglobin parameters in avian myopathies as well. Although near infrared (NIR) spectroscopy has been used for rapid identification of WB fillets in production line^36,37^, no method yet reliably identifies animals susceptible to formation of WB in vivo. We found that sO_2_ prior to the induction of heat stress was higher in chickens that were eventually found to be negative for WB. It is interesting to note that by Day 21, the difference between the two groups had disappeared, with sO_2_ declining in both groups. Because we have shown that such differences in sO_2_ between WB-negative and positive tumors remain consistent over time^20^, it is likely that the lack of difference seen here between the two groups of chickens is due to heat stress.

The average value determined for reduced scattering coefficient (${\upmu }_{\mathrm{s}}{^{\prime}}$) across all wavelengths and animals in our study was about 4 cm^−1^. These values of ${\upmu }_{\mathrm{s}}{^{\prime}}$ are consistent with previous DRS studies that made measurements on freshly excised chicken breast and determined ${\upmu }_{\mathrm{s}}{^{\prime}}$ to be in the range of 3–4.5 cm^−1^ in the same wavelength range^38–40^. These studies found that the absorption coefficient ranged from about 0.4–0.8 cm^−1^ in the wavelength range of 450–600 nm and 0.01–0.04 cm^−1^ at 633 nm. The differences in absorption coefficient (${\upmu }_{\mathrm{a}}{^{\prime}}$) are expected due to the nature of the tissue; these measurements reported in the literature were made on excised breast tissue immediately after excision or after recovery from cold storage while our measurements were performed in vivo, which can lead to substantial changes in hemoglobin concentration, which is the major contributor to absorption coefficient.

In summary, we have shown that diffuse reflectance spectroscopy (DRS) has the potential to dynamically monitor functional changes in the chicken breast in response to environmental stresses. The data presented here indicate that exposure to heat stress results in measurable changes in oxygenation in the chicken breast. Despite this, several questions remain unanswered: (1) What other parts of the chicken experience changes in response to heat stress? (2) What is the minimum level and duration of heat stress exposure that results in measurable changes in oxygenation? (3) Are there other factors that influence these hemodynamic parameters? Controlled studies in a large group of chickens that explore each of these variables could shed light on the ability of DRS to identify a chicken suffering from heat stress through continuous monitoring and thus improve meat quality.
